# The effectiveness of Japanese public funding to generate emerging topics in life science and medicine

**DOI:** 10.1371/journal.pone.0290077

**Published:** 2023-08-17

**Authors:** Ryosuke L. Ohniwa, Kunio Takeyasu, Aiko Hibino

**Affiliations:** 1 Faculty of Medicine, University of Tsukuba, Tsukuba, Japan; 2 College of Medicine, National Taiwan University, Taipei, Taiwan; 3 Center for Biotechnology, National Taiwan University, Taipei, Taiwan; 4 Graduate School of Biostudies, Kyoto University, Kyoto, Japan; 5 Faculty of Humanities and Social Sciences, Hirosaki University, Hirosaki, Japan; Instituto Tecnologico Autonomo de Mexico, MEXICO

## Abstract

Understanding the effectiveness of public funds to generate emerging topics will assist policy makers in promoting innovation. In the present study, we aim to clarify the effectiveness of grants to generate emerging topics in life sciences and medicine since 1991 with regard to Japanese researcher productivity and grants from the Japan Society for the Promotion of Science. To clarify how large grant amounts and which categories are more effective in generating emerging topics from both the PI and investment perspectives, we analyzed awarded PI publications containing emerging keywords (EKs; the elements of emerging topics) before and after funding. Our results demonstrated that, in terms of grant amounts, while PIs tended to generate more EKs with larger grants, the most effective investment from the perspective of investor side was found in the smallest amount range for each PI (less than 5 million JPY /year). Second, in terms of grant categories, we found that grant categories providing smaller amounts for diverse researchers without excellent past performance records were more effective from the investment perspective to generate EK. Our results suggest that offering smaller, widely dispersed grants rather than large, concentrated grants is more effective in promoting the generation of emerging topics in life science and medicine.

## Introduction

Emerging topics (ETs) in basic research, covering emerging technologies, methodologies, issues, and scientific concepts, are reported in scientific articles and become fundamental resources for innovation [1–3]. Meanwhile, in research and development fields, new topics are constantly and cyclically emerging, maturing, converging, and fading out [2, 4, 5]. In the face of such a synergistic and dynamic situation, funding strategies to support efficient generation of ETs, especially successful and high-impact varieties, is critical for policy making.

For industries, outcomes and knowledge from research activities undertaken by universities and public research institutions supported by public funds are an important source of information for both generating R&D and completing existing projects [6, 7]. For example, patents, especially in life science and medicine, tend to cite scientific articles supported by public funds [8, 9]. Here, outside of published articles, the success of industrial innovation based on public scientific outcomes also requires well-managed collaborative research projects and communication between industry and researchers in public institutions [10, 11]. However, scientific articles supported by public funds remain a significant resource for innovation [10, 12, 13].

Diverse studies have reported the effectiveness of public funds on productivity and citation impact of scientific articles as systematically reviewed by Aagaard et al. [14]. A major discussion point in past studies is whether funds should be concentrated only on excellent researchers or be distributed equally among all researchers. In other words, is big science or small science better? In empirical studies, both “too small” and “too large” research grants have been reported as inappropriate to guarantee balanced productivity/impact and funding streams [15–17]. At the same time, the issue of investing solely in researchers with excellent track records remains controversial [18–20].

While studies focusing on citation impact have well demonstrated the association between funds and high-impact research outcomes, the evaluation of associations between funds and generation of novel or emerging topics has been poorly undertaken. This is due to extensive lag between publication and recognition of research articles reporting highly novel or emerging topics [21]. Indeed, articles containing novel topics tend to be produced on the rareness of prior work combinations [22, 23] and tend to appear in lower-impact journals, increasing lag time between publication and citation [24]. However, these articles are eventually cited at a higher rate than articles containing less-novel topics [24]. When we previously compared journal impact factors with the frequency of emerging keywords (elements of ETs) per article in the journal, a slight correlation could be found only in the ranges where impact factors were less than 20 [25]. Thus, any evaluation focusing on articles with high citations over short time periods hardly uncovers the effectiveness of funding on generating novel and ETs over the medium or long term.

Another missing viewpoint in past studies is overall return on investment. Many studies have reported the average or median number of publications/citations per awarded researcher as well as correlations between funding and productivity per awarded researcher [19, 20, 26–33]. While these analyses have clarified the effectiveness of funding on the awarded researcher side, the effect on all researchers remains unclear since it is well known that about 15% to 20% researchers produce 50% of publications, thus ignoring a significant portion of researchers with regard to total investment efficiency [34–37]. Ideally, investor agencies (public and private) need to analyze the total amount of their investment versus the total productivity reported by the awarded researchers as a group while excluding bias generated by a minority of hyperproductive scientists.

In this study, we aim to clarify the effectiveness of public funding on the generation of ETs by analyzing emerging keywords, which are the elements of emerging topics, (EKs; see Conceptual framework), across major academic/industrial fields, in life science and medicine within Japan. We specifically targeted Grants-in-Aid (GiAs) offered by the Japanese Society for the Promotion of Science (JSPS), categorized into life science and medicine-related fields starting between 1991 and 2013 (see Materials and methods). Several previous studies investigated the associations between funding and scientific outputs in Japan, analyzing awarded GiAs [19, 33, 38–40]. However, how the sizes and categories of grants affect the generation of scientific novelty and impact are still poorly addressed. In addition, overall return on investment is rarely mentioned in published studies. Thus, in this study, we investigated the effectiveness of these funds on the generation of EKs. Furthermore, we included highly successful emerging keywords (HS-EKs; see Materials and methods) by comparing the number of EKs reported by the awarded PI before and after the start of funding (EK and HS-EKs reported from 1988 to 2018). At the same time, we also analyzed the overall effectiveness of funding on the generation of EKs and generated differential conclusions from the viewpoints of both PI and investor sides.

## Conceptual framework; emerging keywords as elements of emerging topics

Scientometric publications centered on ETs research have increased notably within the last 10 years and are now generating interest in policy circles [2, 41, 42] as reported in Science and Technology Studies [43, 44]. The study of ETs requires a rigorous definition, provided by Rotolo et al. (2015), in which the operational definition of emergence is defined by scientometric methodologies grouped into 5 main categories: 1) indicators and trend analysis, 2) citations analysis, 3) co-word analysis, 4) overlay mapping, and 5) combinations of these methodologies. Recent studies by Xu et al. (2019) and other research groups have shown a shift from citation-based to machine learning-based approaches in the methodologies used for analyzing emerging topics to predict future emerging topics [45–47]. However. a crucial challenge is to develop a method for identifying emerging topics at their early stages, without machine learning-based analyses, to clarify the role of investment for fostering scientific advancements and novel technologies [48, 49].

**Fig 1:**
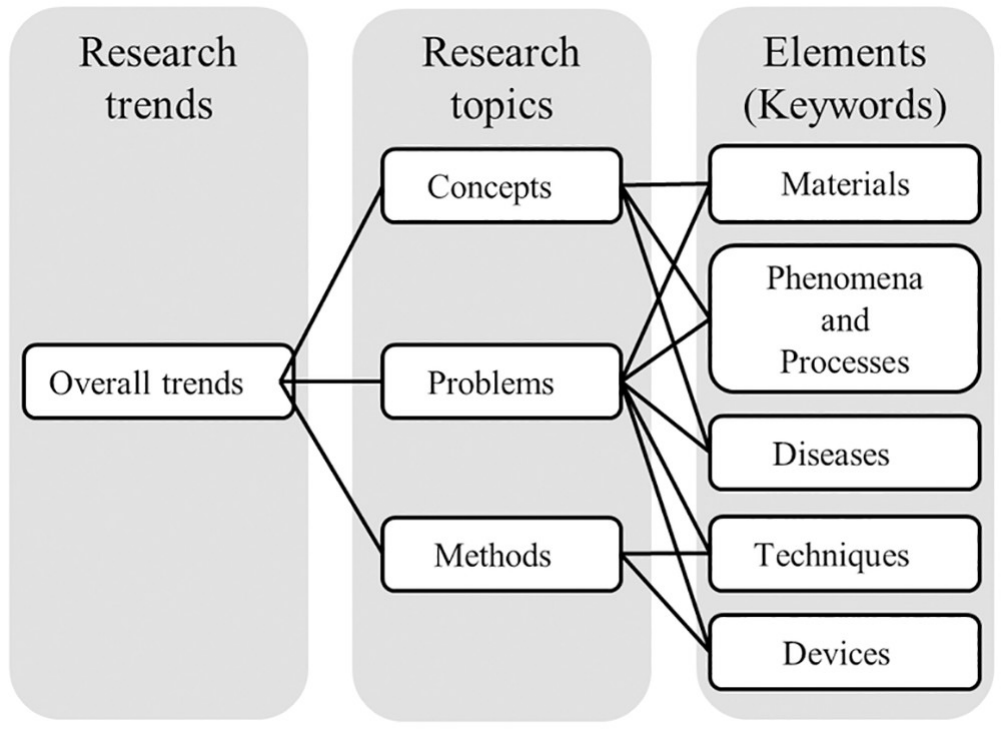
From keywords to trends.

We previously demonstrated that the retrospective study with co-word analysis for vast datasets was still effective to comprehend the mechanisms that generate emerging topics [4, 50, 51]. Our in-house scientometrical method for quantifying past and current ETs in life science and medicine via PubMed, currently the main repository for these types of articles [5], was listed as a representative method for co-word analysis by Rotolo et al. [2]. For ETs, scientific specialties, first reported by Braam et al. [52], are defined by discreet sets of subject-related issues, problems, methods, and concepts that draw focus from researchers regardless of background (Fig 1). In this manner, research topics become an aggregation of specific keywords that best represent those specific topics.

With this model, we previously defined ‘Emerging Keywords (EKs)’ from the Medical Subject Headings (MeSH) [53] attached to PubMed articles as terms included in the top 5% by incremental rate in a given year [5]. With this operation, we can select MeSH terms as EKs which should be considered emergent at a particular time in the past. We can then finally cluster EKs that co-appeared in the same articles to generate ETs [5]. Our definition to identify emerging topics is frequently used [41, 45, 54, 55].

**Fig 2:**
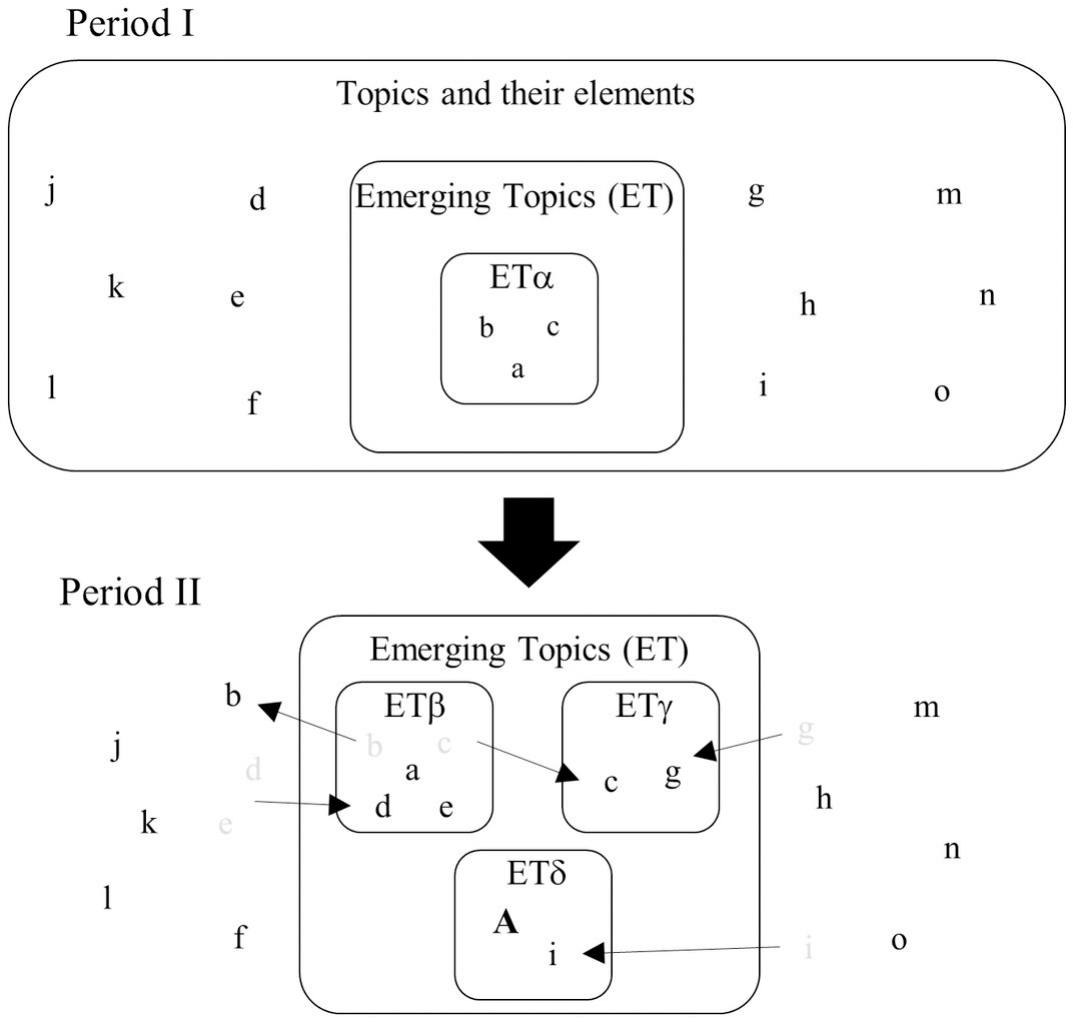
Schematic model of the generation process of ETs, modified from Ohniwa et al. 2019 [4]. Lowercase letters (a to o) indicate the elements of life science topics that are represented by MeSH terms. Uppercase letters (A and B) indicate the newly appeared elements of new ETs. ETα to ETζ represent distinct ETs. Each ET is composed of various elements representing materials, phenomena and processes, diseases, techniques, devices, etc. A certain ET in a certain period evolves into a different ET by dropping and acquiring different elements over time. For example, ETα in Period I changes into ETβ in Period II by dropping off elements b and c and acquiring elements d and e, which are not the elements of ETs in Period I. Certain elements may also recruit other elements to generate new ETs. Element c in ETα in Period I recruits element g to generate distinct ETγ in Period II, for instance. Newly appeared elements also sometimes generate novel ETs. For example, in Period II, element A appears and generates ETδ together with existing element i.

By using the number of EKs as an indicator, it becomes possible to assess the level of developing new ideas. The accumulation of new EKs together with dropping pre-existing EKs from the keyword clusters finally generates new ETs (Fig 2), with more than 70% of total EKs generated in this manner [4]. EK is a component of ET and, by using the number of EKs as an indicator in this study, it becomes possible to specifically assess where new ideas are developed and who develops them.

Additionally, by tracking the temporal changes of EKs, we can quickly grasp changes in technology and research subjects related to innovation from changes in topic elements such as subject-related materials, phenomena, techniques, devices, etc. (Fig 1). For instance, the advent of the post-genomic era after 2000 due to the development of large-scale analysis techniques in the life sciences and medical science fields is well-known to have been a significant innovation. By utilizing our EK tracking method, we clarified that this movement was already happening in the mid-1990s [4]. We also successfully grasped the development of nanotechnology and the advancement of RNA technology in the respective fields at their early stages [5]. There, we can trace the trend from the co-occurrence of EKs in a few papers at the early stages of topic development (the budding stage of the topic by a few researchers) to the co-occurrence of the same EKs in many papers as a result of topic development (its spread among researchers).

In summary, this keyword-based approach is more granular than citation-based metrics as it allows for the identification of specific keywords that are associated with innovation. This approach also has an advantage in its timeliness to capture the early stages of innovation. Finally, by using a keyword-based approach, we can capture the process from the formation of ET at the initial budding stage to igniting and establishing innovation in the research community at the level of topic elements.

## Materials and methods

### JSPS grants and PI datasets

The Japan Society for the Promotion of Science (JSPS), now supervised by the Ministry of Education, Culture, Sports, Science and Technology (MEXT), has been offering competitive and peer-review based GiAs for decades to all types of scientific researches in Japan. The original GiAs were initiated in 1939 and remain the largest source for public curiosity-driven science grants in Japan covering all academic fields from humanities and social sciences to the natural sciences [33, 38, 56]. Notably, GiAs weight basic research more heavily than applied ones (National Institute of Science & Technology Policy 2004). Researchers belonging to institutions officially approved by MEXT can apply for GiAs as Principal Investigators (PI) and these awards include both direct costs paid to promote research activities and indirect costs paid to the institutions to support research activities. Unlike grants in US, the GiAs do not cover salaries of the PIs themselves but they can pay personnel expenses for researchers and staff.

GiAs offer diverse curiosity-driven funding programs according to project purpose and targeted researchers by manipulation of the grant categories. For example, GiAs for Scientific Research types (A), (B), and (C) were initiated in 1996 to target creative/pioneering research conducted by one researcher or jointly by multiple researchers. Currently, type (A) is for 3 to 5 years with 20 million to 50 million yen total, type (B) is for 3 to 5 years with 5 million to 20 million yen total, and type (C) 3 to 5 years with 5 million yen or less total (https://www.jsps.go.jp/english/e-grants/grants01.html). Here, if subcategories exist within the same category, the applicants are filtered through a selective merit process that increases in difficulty with award amount. From 2001, a new category with larger grant sizes (50 million to 200 million yen total), the GiA for Scientific Research (S), was additionally started for supporting creative/pioneering research conducted by one or a relatively small number of researchers. In 2003, with the purpose to support young and early-career researchers, GiAs for Young Scientists was initiated (currently renamed GiA for Early-Career Scientists with the modification of its qualification requirements). The GiA for COE Research, which existed from 1995 to 2005, offered over 1 billion yen per project, aiming to cultivate a competitive academic environment among Japanese universities by providing targeted support for the creation of world-class research and education bases. The basic information of all GiAs investigated in this study is summarized in S1 and S2 Tables with the acceptance proportions indicated.

JSPS grant information was retrieved from the KAKEN Grant Database of Grant-in-Aid for Scientific Research (https://kaken.nii.ac.jp/en/index/) web site on March 19^th^, 2021. We collected grants whose projects started between 1991 and 2013 and were categorized into a specific Research Category listed in S1 Table. To restrict the grants to life science and medicine-related fields, we selected grant information categorized in specific Research Fields on the Database as shown in S3 Table. Since, among the grants, GiA for COE Research was not classified into any Research Fields, we manually selected only grants related to life science and medicine.

The PI name and affiliation in English for each awarded grant were retrieved from the KAKEN Researcher Database of Grant-in-Aid for Scientific Research web site (https://nrid.nii.ac.jp/en/index/) by using researcher IDs originating from the retrieved grant information in the KAKEN Grants site on January 1st, 2022 since the KAKEN Grants website frequently lists names and affiliations only in Japanese. From the retrieved grant data set, we excluded any grant information without English PI names and affiliation in both KAKEN Grant and KAKEN Researcher databases. By this operation, we selected 182,810 grants out of 209,732 grants collected in the above paragraph.

### Article and associated MeSH terms dataset

Medical Subject Headings (MeSH) is a popular keyword database developed by the US National Library of Medicine. Content-specific MeSH headings are attached to each article under the supervision of professional curators and typically used by PubMed to support literature searches [53]. MeSH terms attached to articles published between 1987 and 2020 were collected from PubMed (https://www.ncbi.nlm.nih.gov/pubmed/) on December 8th, 2021 and included a total of 26,061,316 articles for analysis. We applied the 2021 version of the MeSH tree structure information to analyze the hierarchy of MeSH terms. Details on MeSH term identification attachment can be found in our previous reports [4, 5]. This operation obtained 256,680 kinds of terms, totaling 1,190,037,134 occurrences spanning from 1987 to 2020.

### Emerging keywords and highly successful emerging keywords

The method to identify EKs from MeSH terms was previously reported [5]. Briefly, the increment rate (I) of MeSH term n in year t was calculated as:

$${\text{I}}_{\text{n}}{}_{\text{in}\;\text{t}}={\text{X}}_{\text{n}}{}_{\text{in}\;\text{t}}/{\text{Y}}_{\text{n}}{}_{\text{in}\;\text{t}}$$

where X_n in t_ is the total number of appearances of MeSH term n on PubMed in years t+1 and t+2, and Y_n in t_ is the total number of appearances of MeSH term n in years t-1, t, t+1 and t+2. EKs were defined as the terms ranked in the top 5% of I_n in t_ in year t.

In the present study, when a particular MeSH term was counted, those terms located in the higher positions of the hierarchy were additionally counted since higher-level terms are inclusive of lower-level terms. We thus collected 97,864 kinds of MeSH terms as EKs, totaling 48,211,167 occurrences that spanned the years 1988 to 2018.

An HS-EK was previously defined as satisfying the following criteria: 1) the number of its appearances after 10 years of being designated an EK is at least 10 times larger than its initial year and 2) the total number of its appearances after 10 years is more than 100 [4, 51]. While this criterion is arbitrarily set, in practice it allows us to obtain various MeSH terms related to Nobel Prize-winning topics, such as ‘Oncogene Proteins, Viral’ in 1980 for the Nobel prize-winning topic of “the cellular origin of retroviral oncogenes” in 1989 and ‘Apoptosis’ in 1991 for “genetic regulation of organ development and programmed cell death” in 2002 [4]. Using these criteria, we identified 3,556 kinds of highly successful emerging keywords out of 225,485 kinds of emerging keywords that appeared from 1989 to 2010.

### Identification of articles published by grant-awarded PIs

In PubMed, author names frequently appear as family name plus initials of the middle and last names (e.g., Ohniwa RL) and affiliations, such as institutions, were mostly attached to only first or corresponding authors in PubMed before 2014 [51]. In this study, we identified articles published by a particular PI as ones that included family name and surname initials, plus the initials of middle and/or additional names, and institution name in the same article. Thus, we accepted the risk of counting different authors as the same authors. However, since corresponding authors are usually the PIs and first authors usually belong to the PI’s lab, we considered this better than exhaustive analyses to determine perfect research institution matches. Our previous exhaustive study on researcher dynamics to generate ETs on PubMed showed that the identification of articles using only author name (family name plus the initials of middle and/or last names) demonstrated the same tendencies as full affiliation results after 2015 [51], supporting our criterion for the current exhaustive analyses.

### Effectiveness of investment to generate emerging topics

To inclusively evaluate the overall effectiveness of a particular grant to contribute to ET generation, we introduced the following equation to estimate the Effectiveness of Investment (EI) of a specific grant category g (EI_g_):

$${\text{EI}}_{\text{g}}=\left({\text{E}}_{\text{g}}+100{\;\text{x}\;\text{H}}_{\text{g}}\right)/{\text{I}}_{\text{g}}$$

where, E is the total number of EKs reported by the awarded PIs in the six years after the grant g began, H is the total number of HS-EKs reported by the awarded PIs in the six years after the grant g began, and I is the total invested amount in grant category g. Here, we set the following aspect for the equation; about one in one hundred EKs can be picked up as HS-EKs (In the case from 1991 to 2004, total EK numbers were 17,493,847 while total occurrence of HS-EKs was 189,460. Thus, 17,493,847/189,460 = 92.3).

## Results and discussion

### Relatively small grants more effectively generate articles and emerging keywords

Our aim was to evaluate the effectiveness of Grants-in-Aid (GiAs) offered by JSPS with special attention to the generation of ETs in life science and medicine fields. As such, we analyzed the number of EKs (elements of ETs) reported by the awarded PIs before and after the start of funding cycles. We therefore targeted all grants beginning between 1991 and 2013 as listed in S1 Table and compared the number of EKs with the number of articles reported by the PIs three years before and six years after the start of funding.

**Fig 3:**
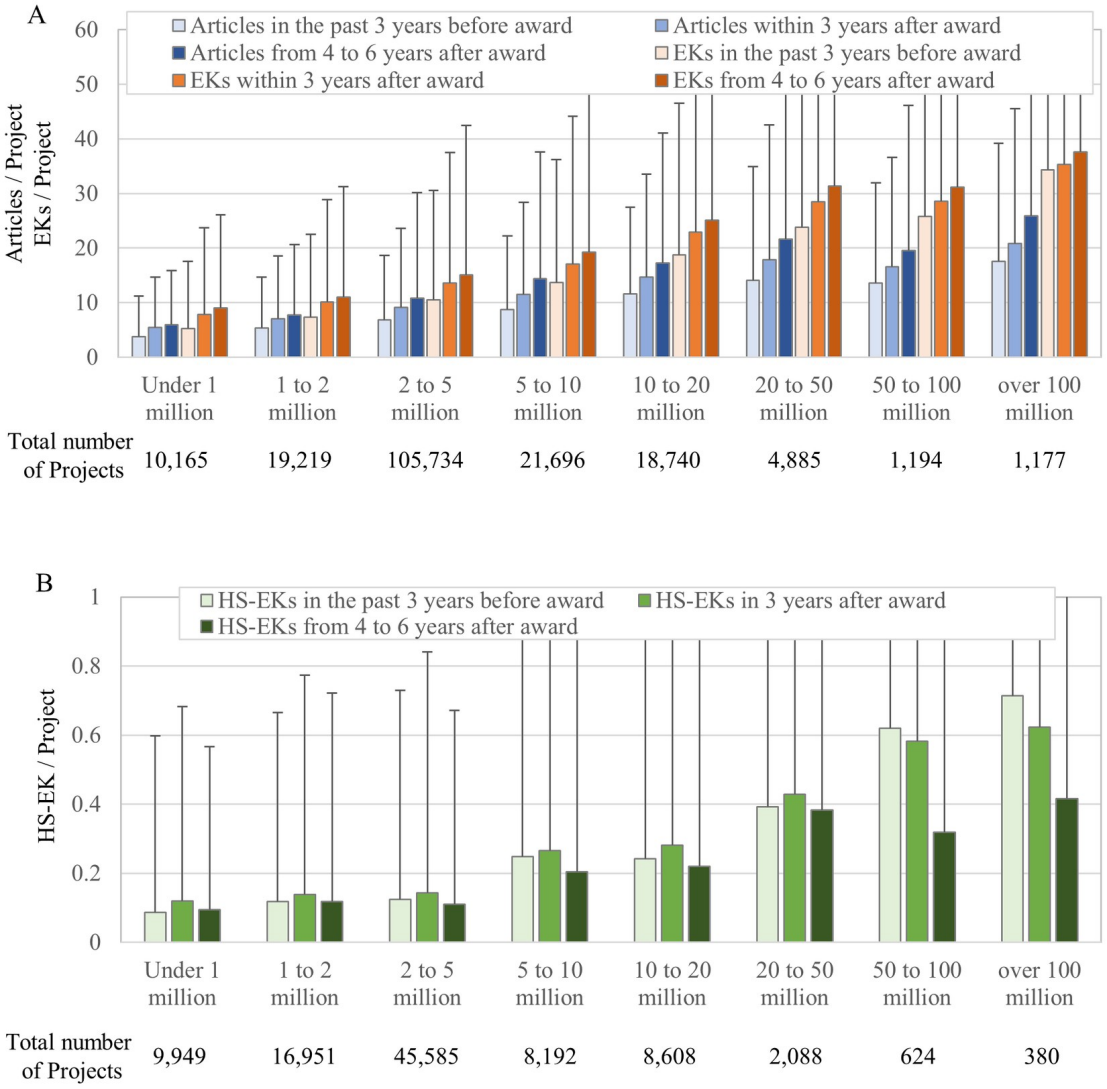
Average number of reported articles, EKs, and HS-EK per PI of research project according to grant size range. (A) The number of published articles and EKs in the past 3 years before funding began, within 3 years after funding began, and within 4 to 6 years after funding began. The data includes grants beginning from 1991 to 2013. When comparing the values of the before and after periods within each category, both the number of research papers and the number of EKs show p < 0.01. (B) The number of published HS-EKs in the past 3 years before funding began, within 3 years after funding began, and within 4 to 6 years after funding began. The data includes grants beginning from 1991 to 2004. (A)(B) X and Y axes show the range of grant amount and averages with standard deviations, respectively. All amounts are in Japanese yen.

First, we calculated the average numbers of EKs in articles published by the awarded PIs across the range of the total amount of grants (Fig 3A). In all amount ranges, the average numbers of both published EKs and articles increased within three years after the funding began and again after four to six years. Here, higher amounts (especially ranging up to 50 million JPY and over 100 million JPY) correlated with higher EK and article production after the funding began. Although there were no differences in the average number of EKs generated by grants between 20 to 50 million JPY and 50 to 100 million JPY (where the average number of articles is even less than 20 to 50 million JPY), it is likely that PIs receiving more money will generate more articles and EKs.

**Fig 4:**
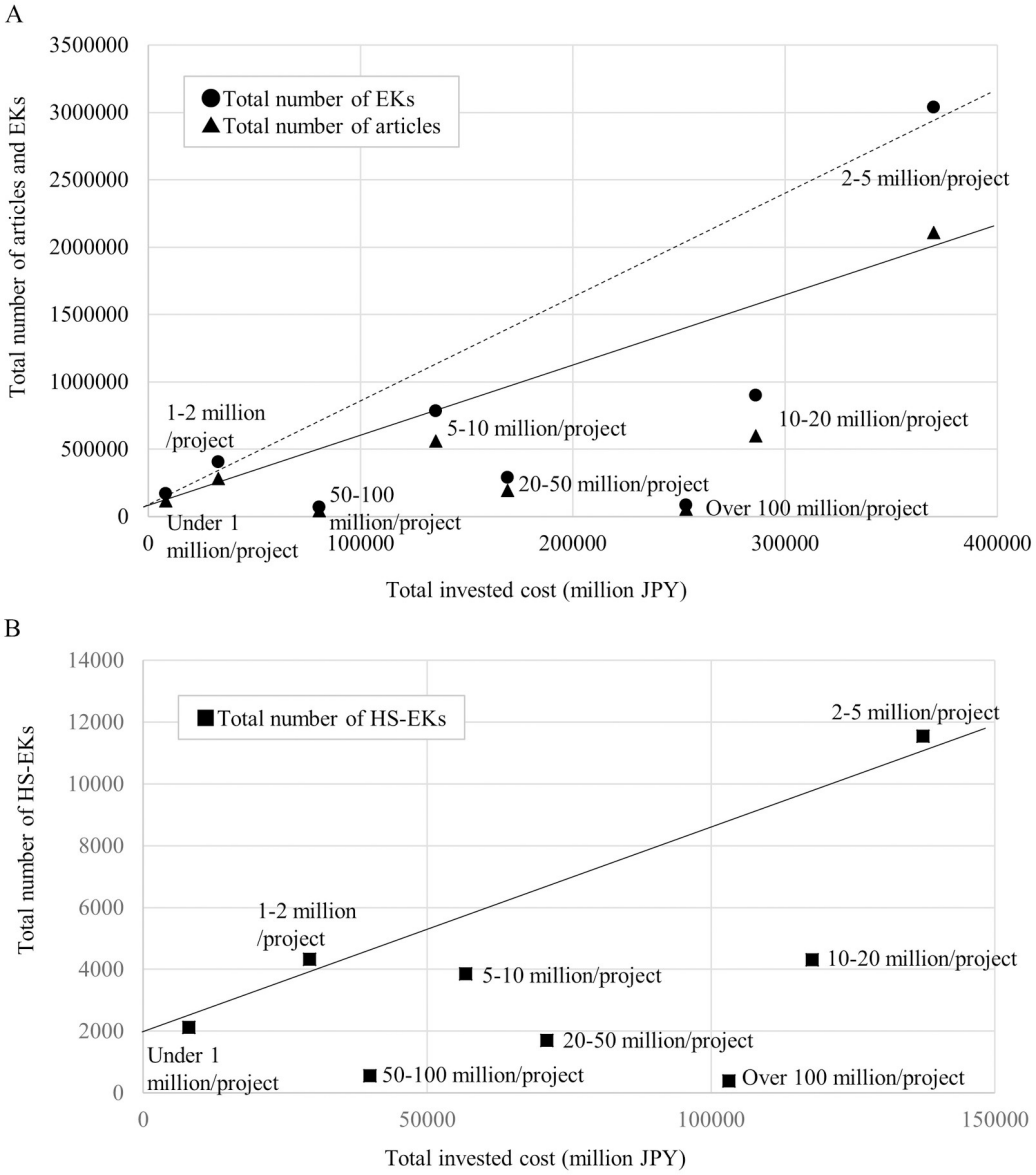
Total invested cost by JSPS vs total number of articles, EKs, and HS-EKs reported by PIs. (A) Total number of published articles and EKs within 6 years after funding began. The data includes grants beginning from 1991 to 2013. X and Y axes show total invested cost and total number of reported articles and EKs. Solid and dashed lines represent linear fit from under 1 million JPY/project, 1–2 million JPY/project and 2–5 million JPY/project of the number of published articles and EKs, respectively. (B) Total number of published HS-EKs within 6 years after funding began is shown. The data includes grants beginning from 1991 to 2004. X and Y axes show total invested cost and total number of reported HS-EKs. Solid lines represent linear fit from under 1 million JPY/project, 1–2 million JPY/project and 2–5 million JPY/project of the number of published HS-EKs. All amounts are in Japanese yen.

However, this trend was not exponential. Since doubling grant money did not double EK production (Fig 3), investors should evaluate the effectiveness of the total amount of investment on EK and article production. Fig 4A shows the total investment in each amount range versus the total number of EKs and articles published within six years after the funding began. While there is a linear relationship between the numbers of publications and accompanying EKs in the ranges up to 5 million JPY, this linearity is lost above 5 million JPY. These results suggest a “too-large” cutoff, beyond which grants are not effective for EK generation, and that grants of less than 5 million JPY are more effective for Japan.

### The relationship between total investment amounts and the production of highly successful emerging keywords

ETs, in general, are expected to influence scientific and public societies with impact and innovation. However, we previously demonstrated that many EKs (the core element of ETs) fade away without any appreciable influence [4]. Therefore, it is valuable to analyze funding effectiveness at generating successful EKs. HS-EKs are identified by including various MeSH terms related to Nobel Prize-winning topics [4, 51] and, thus, represent elements of high-impact ETs.

Our study focused on grants which began between 1991 and 2004 for HS-EKs and tracked HS-EKs for 6 years (until 2010). Unlike the EKs shown in Fig 3A, the average HS-EK generation over three years increased only up to the grant amount range of 50 million JPY and actually decreased above 50 million JPY (Fig 3B). Here, the average numbers of HS-EK generation in all amount ranges were less four to six years after funding started (Fig 3B). Therefore, it is likely that, for further generation of HS-EKs, PIs are better off receiving smaller grants (less than 50 million JPY) over short periods.

To evaluate the effectiveness from the investor side, we compared the relationship between the total amount of investment and the total number of generated HS-EKs within six years after funding began (Fig 4B). Like the generation of EKs and articles, a good linear fit was found over ranges up to 5 million JPY, but no higher, where higher amounts created more divergence from the line of best fit. Thus, for the generation of HS-EKs, grant amounts of less than 5 million JPY are effective from an investor standpoint.

### The productivity of articles, EKs, and HS-EKs in different grant categories

**Fig 5:**
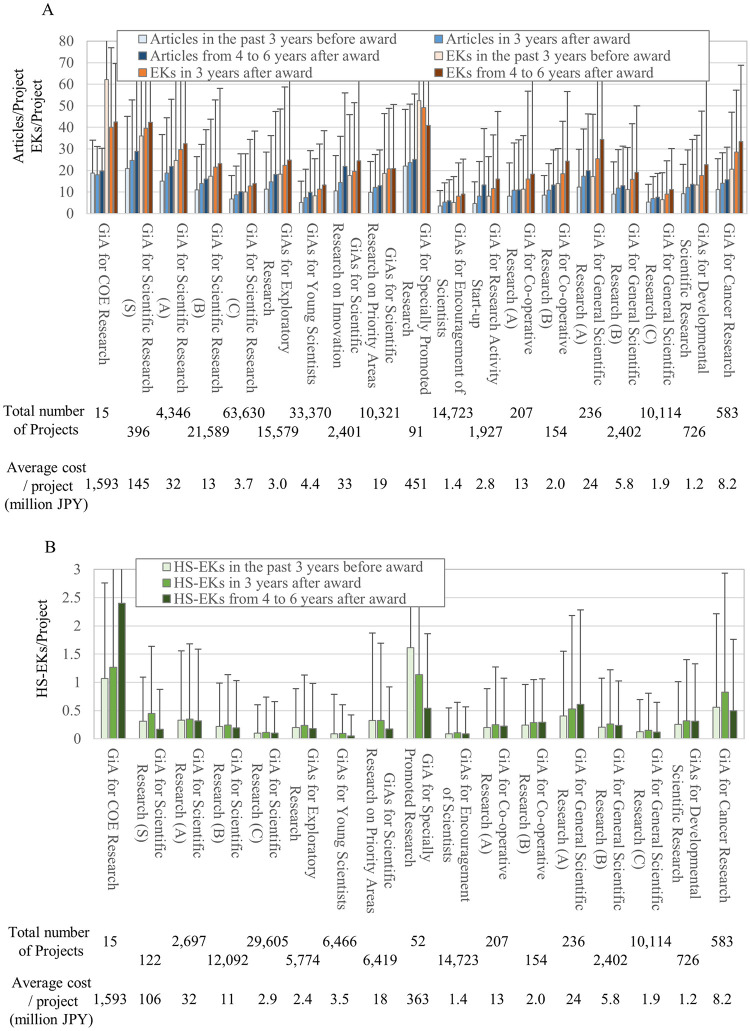
Average number of reported articles, EKs, and HS-EK per PI of research project in each GiA category. (A) The number of published articles and EKs in the past 3 years before funding began, within 3 years after funding began, and within 4 to 6 years after funding began. The data includes grants beginning from 1991 to 2013. (B) The number of published HS-EKs in the past 3 years before funding began, within 3 years after funding began, and within 4 to 6 years after funding began. The data includes grants beginning from 1991 to 2004. (A)(B) X and Y axes show the range of grant amount and averages with standard deviations, respectively. All amounts are in Japanese yen.

In Japan, each JSPS grant category has its own purpose (e.g., concentrated on excellent researchers, distributed equally, encouraging start-ups, supporting continuous research, or given only to young scientists, etc.), regardless of its budget size. Consequently, we next evaluated the effectiveness of each grant category to generate articles, EKs, and HS-EKs by the awarded PIs. Fig 5 shows the average number of articles, EKs, and HS-EKs generated in each grant category. In the case of articles and EKs, except for GiA for COE Research and GiA for Specially Promoted Research, the total number of EKs and articles increased after the funding began in three years and further increased within three to six years. In contrast, for HS-EKs, such tendencies were only found in GiA for COE Research and GiA for General Scientific Research (A), with most other GiAs showing their highest HS-EK productivity within three years after the funding began. On the contrary, the productivity of HS-EKs in GiA for Specially Promoted Research was the highest before the funding begun. This HS-EK trend might imply that the research projects proposed by each PI in the grant, consisting of potentially high-impact topics, are concluded and resulted in published papers within three years after the funding initiation. It also suggests a potential inability of the PIs to generate new high-impact topics beyond that timeframe.

**Fig 6:**
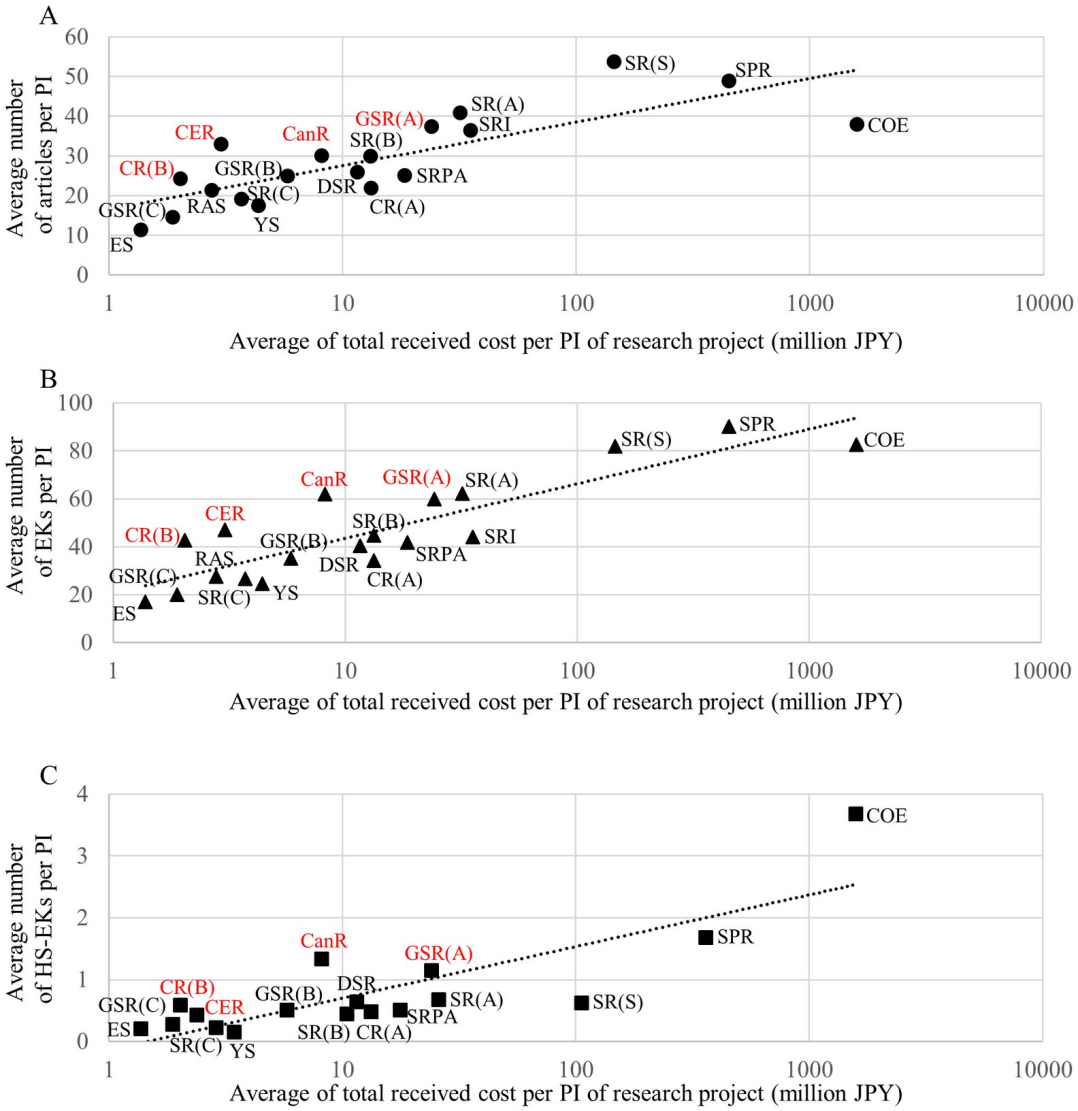
Average of total received cost per PI of each research project vs. average number of articles, EKs, and HS-EKs reported by the PI. The X axis shows average number of published (A) articles, (B) EKs and (C) HS-EKs within 6 years after funding began and the Y axis shows the average of total received cost per PI of each research project (millions JPY). The data includes grants beginning from (A)(B) 1991 to 2013, and (C)1991 to 2004. Dashed lines represent linear fit from all the points. Abbreviations represent the following. COE; GiA for COE research, SR(S); GiA for Scientific Research (S), SR(A); GiA for Scientific Research (A), SR(B); GiA for Scientific Research (B), SR(C); GiA for Scientific Research (C), CER; GiAs for Exploratory Research, YS; GiAs for Young Scientists, SRI; GiAs for Scientific Research on Innovation, SRPA; GiAs for Scientific Research on Priority Areas, SPR; GiA for Specially Promoted Research, ES; GiAs for Encouragement of Scientists, RAS; GiA for Research Activity Start-up, CR(A); GiA for Co-operative Research (A), CR(B); GiA for Co-operative Research (B), GSR(A); GiA for General Scientific Research (A), GSR(B); GiA for General Scientific Research (B), GSR(C); GiA for General Scientific Research (C), DSR; GiAs for Development Scientific Research, CR; GiA for Cancer Research.

To investigate which particular grant categories are more productive in the generation of articles, EKs, and HS-EKs after grant funding, we plotted the average grant amount received by the PIs against the average numbers of articles, EKs, and HS-EKs within six years after the funding began (Fig 6). Linear fittings of all the categories showed that GiA for Co-operative Research (B), GiAs for Exploratory Research, GiA for Cancer Research, and GiA for General Scientific Research (A) appeared in the upper regions of the fit lines for articles, EKs, and HS-EKs. It is likely that these grant categories were relatively successful in generating output from funding.

**Fig 7:**
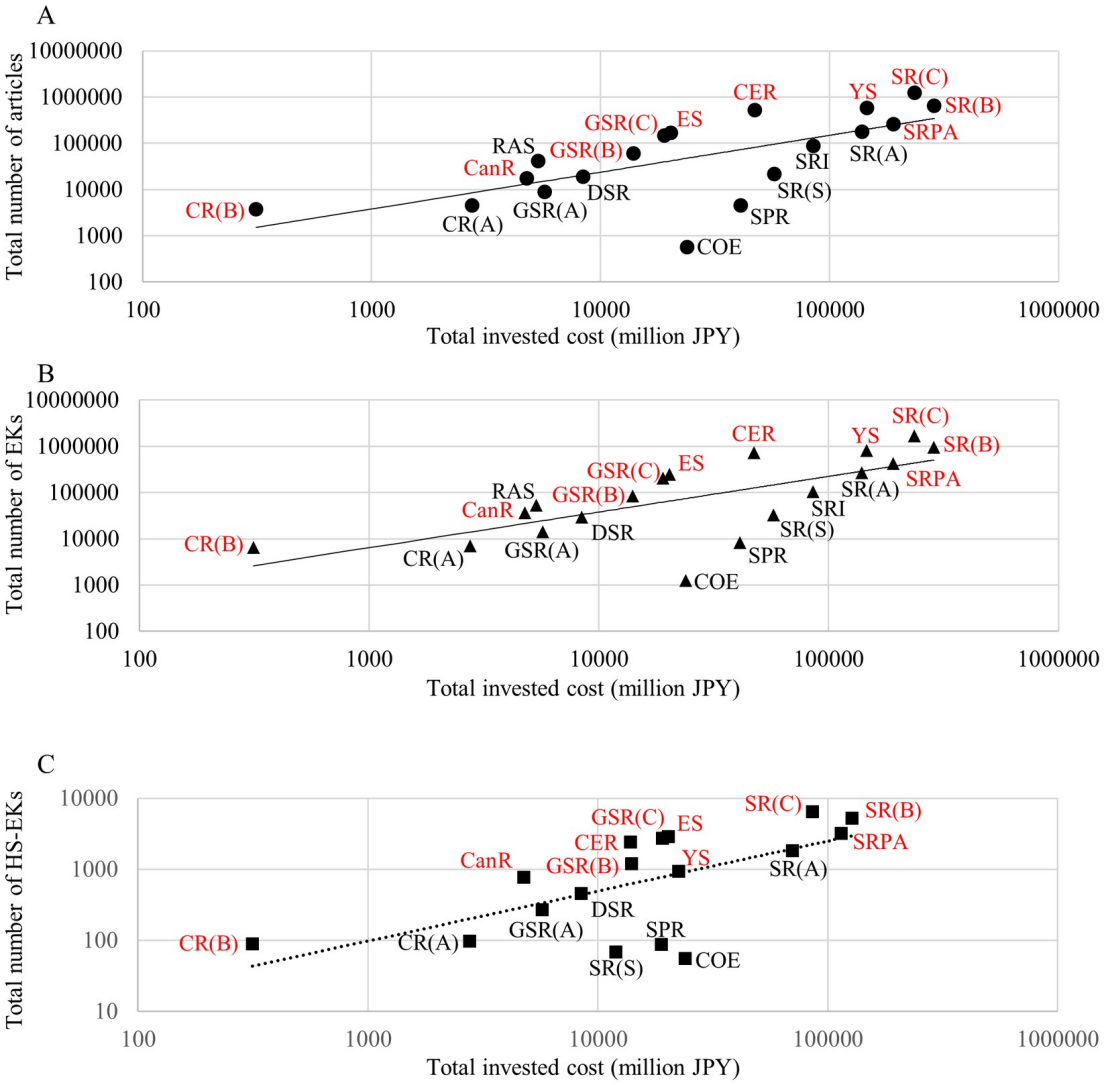
Total invested cost by JSPS vs. total number of reported articles, EKs, and HS-EKs by all PIs of a particular grant category. The X axis shows total number of published (A) articles, (B) EKs, and (C) HS-EKs within 6 years after funding began and the Y axis shows total invested costs (millions JPY). The data includes grants beginning from (A)(B) 1991 to 2013, and (C)1991 to 2004. Dashed lines represent linear fit from all the points. Abbreviations are the same as Fig 6.

To evaluate investment efficiency, we also compared the total invested amount in each grant category and the total number of articles, EKs, and HS-EKs generated within six years by the awarded PIs (Fig 7). The linear fittings demonstrated that GiA for Co-operative Research (B), GiA for Cancer Research, GiA for General Scientific Research (B), GiA for General Scientific Research (C), GiAs for Encouragement of Scientists, GiAs for Exploratory Research, GiA for Scientific Research (B), GiA for Scientific Research (C), GiAs for Young Scientists and GiAs for Scientific Research on Priority Areas appeared in the upper region of the fit line. Relatively small grants were again more productive. Together with the result of the average (Fig 6), GiA for Co-operative Research (B), GiA for Cancer Research, and GiAs for Exploratory Research also seemed to be comparatively successful grant categories in terms of the effort balance between investors and PIs.

### Grant distribution of moderate amount categories to a variety of researchers is important

In order to determine research policy, consideration of investment effectiveness on various aspects of research productivity is important. In the above analyses, we separately evaluated grant effectiveness to generate EKs and HS-EKs. To inclusively evaluate which category is the most effective at generating emerging topics from the investor side, we introduced a simple equation to assess the effectiveness of the total investment on scientific outcomes related to the generation of emerging topics (EIs; see Materials and methods).

**Fig 8:**
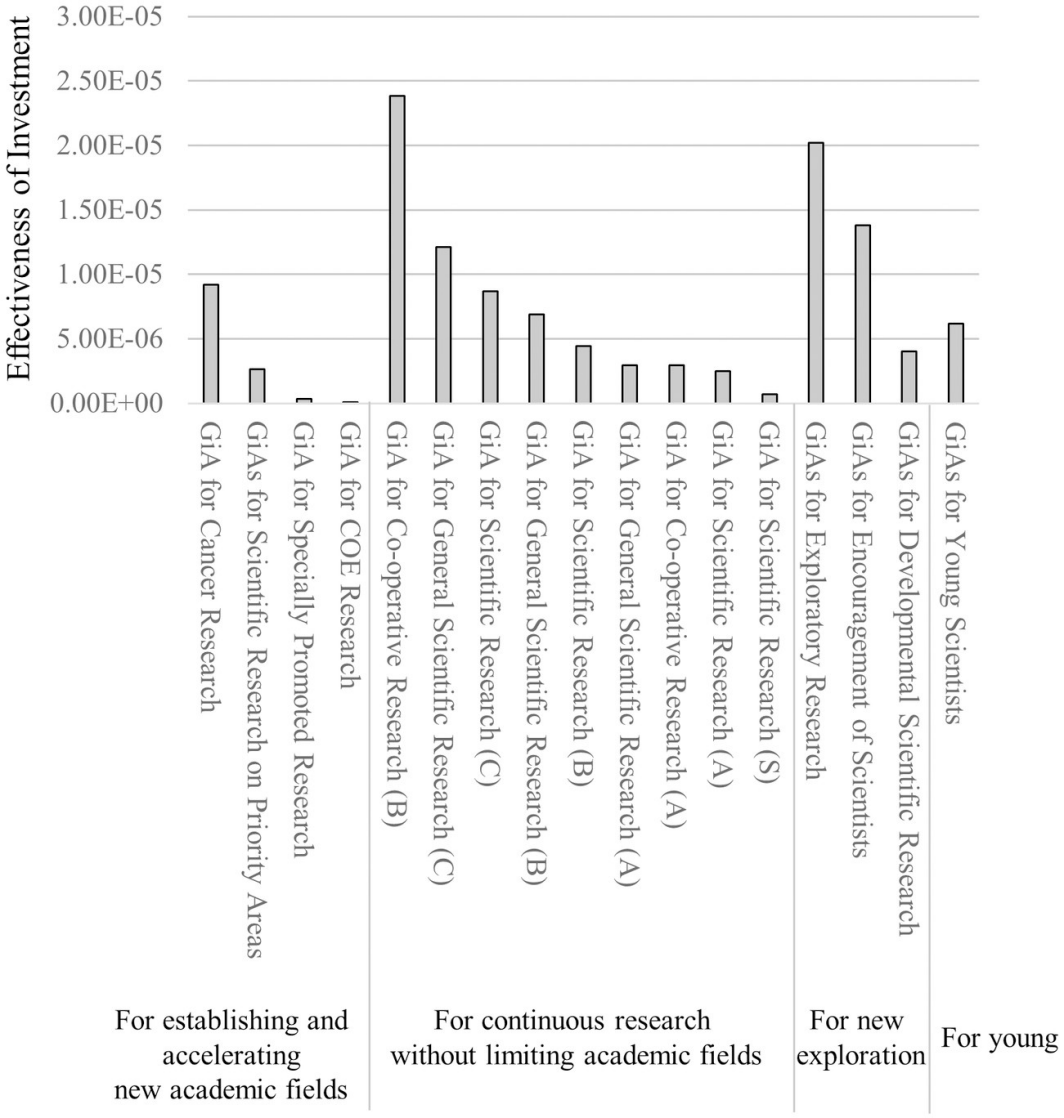
Effectiveness of Investment (EI) to generate emerging topics estimated by data from 1991 to 2004.

The result demonstrated that the best grant category was GiA for Co-operative Research (B), followed by GiA for Challenging Exploratory or Exploratory Research, GiA for Encouragement of Scientists, GiA for General Scientific Research (C), and GiA for Scientific Research (C) (Fig 8). The sizes of these grants are relatively small; each is less than 5 million JPY total over three years (S1 Table). This is consistent with our results on the amount range of the grants (Figs 3 and 4). Of note, these grant categories did not target only researchers with excellent publication records. As shown in Fig 3, PIs receiving relatively small grants generated smaller numbers of published articles, EKs, and HS-EKs before staring the funded project. Thus, for the investor side in Japan, moderate, short-term grant distribution to a wide variety of researchers is likely a more effective strategy to promote the generation of ETs.

When categorizing each grant into research funding types aimed at establishing and accelerating a new academic field, continuous research without limiting the field, and/or categories emphasizing new exploration, it is observed that each classification includes both relatively high and low EI values (Fig 8). This suggests that the funding amount has a greater influence than the characteristics of the grant category.

## Conclusion and implications

### Smaller grants to many researchers are preferable in Japan

In this study, we evaluated the effectiveness of grants to generate ETs by analyzing the articles, EKs, and HS-EKs reported by the awarded PIs. Both the amount range- and category-dependent analyses demonstrated that grants of less than 5 million JPY are more effective at generating ETs. Since the average number of published articles, EKs, and HS-EKs became progressively smaller in line with grant budget size (Fig 3), such small grants were not concentrated only on researchers with past excellent records. Although competitive (acceptance rates varied from 10% to 30%) (S2 Table), good proposals had higher chances to receive funding even if they were not submitted by top-tier researchers.

In this study, we focused on grants from the 1990s to the 2010s but there were none that persisted throughout the entire period. It is particularly well known in life sciences and medicine that there has been a significant shift in research approaches between the pre-2000s and post-2000/post-genomic era. We previously reported that post-genomic era research requires more manpower plus focused and sustained effort [5, 51]. We also demonstrated a mode-shift within the scientific culture of life science and medicine to generate ETs before and after the genomic era, i.e., the “progressive stage” with fruitful, novel findings facilitated by identification and manipulation of genes until the late 1990s. This contrasts with the “re-evaluation stage,” overlapping with the modern “post-genomic era,” that focuses more on re-analyzing old topics by leveraging newly developed methods such as computational techniques, large-scale analyses, and nano-scale analyses [5, 51]). Some of our targeted grants existed only till the mid-1990s (e.g., GiA for Co-operative Research [B] and GiA for General Scientific Research [C]) while others came into being only after the mid-1990s (e.g., GiA for Scientific Research [C]). However, regardless of the mode-shift, smaller grants were ranked in the top 5 performers of our EI evaluation (Fig 8). Therefore, we conclude that smaller grants distributed to many researchers without excellent performance records are the most effective to promote the generation of ETs. Taken together, our analysis indicates that “small” science is better in life science and medicine research from the investor or policy maker viewpoint in Japan.

### Other implications for research policy in Japan

Another implication is the effectiveness of funding for the awarded PIs to generate ETs. In this study, PIs receiving larger grants generated more articles, EKs, and HS-EKs after the grants began (Fig 3). In this case, PIs capturing higher grant funding tended to have excellent past performance at generating articles, EKs, and ETs (Fig 3). Thus, if the investor side can ignore the overall return on the investment, more funding to PIs with better performance records can facilitate the generation of more ETs. In this regard, “big science” does have a place in life science and medicine from the standpoint of supporting productive PIs in Japan.

Public support to young and/or early career researchers is considered a keystone factor in their future performance and careers. A dataset analysis from the Netherlands showed that the number of publications by receivers of early career grants was slightly higher than non-receivers but the citation impact of publications between the groups was similar, especially regarding high citation articles [57]. In the case of Japan, when focusing on individual PI performance, competitive project funding for early career researchers led to lower generation of novelty than block funding [40]. In our results, the average number of articles, EKs, and HS-EKs per young/early-career PI (GiAs for Young Scientists, GiAs for Encouragement of Scientists and GiA for Research Activity Start-up) tended to be relatively low compared with the other GiAs (under the average line of fit, excepting HS-EKs for GiA for Research Activity Start-up) (Fig 6). In contrast, the overall return on investments of these grants to generate publications, EKs, and HS-EKs were relatively high (over the line of fit) (Fig 7). The EI rankings of GiAs for Young Scientists and GiAs for Encouragement of Scientists were 8 and 3 out of 17 grant categories, respectively (Fig 8) (GiA for Research Activity Start-up was excluded because of its 2010 start date). Thus, from an investor viewpoint only, support to young and/or early career researchers in Japan seems to be functioning.

### Concluding remarks and possible future research

Our results demonstrated that, in terms of grant amounts, while PIs tended to generate more EKs with larger grants, the most effective investment from the perspective of investor side was found in the smallest amount range for each PI. It is noteworthy that this study clarified the asymmetric structure between individual PI perspective and investor side with respect to effectiveness of grant concentration. Second, in terms of grant categories, we found that grant categories providing smaller amounts for diverse researchers without excellent past performance records were more effective from the investment perspective to generate EKs. This result emphasized the importance of diversity for generation of scientific novelty. It might be possible that smaller grants allow researchers to take more risks, explore new ideas, and help build relationships with other researchers to get their work noticed. Another interpretation is that pools of many smaller grants could foster greater diversity of research objects, materials, and methods. Investigations of these small-grant researchers will be valuable to clarify the reason why smaller grants are more effective.

In this study, we restricted our analysis to awarded PIs to isolate results from effects due to co-investigators for the same grants and additionally excluded grant-funded researchers, including postdocs, project-based faculty members, technicians, etc. In general, since larger grants tend to include more researchers in separated groups, if the number of publications, EKs, and HS-EKs reported by researchers other than PIs in a particular grant is counted, assessments could be biased, especially in the case of larger grants. This point is crucial for future studies to evaluate the true effectiveness of science funding.

This study only analyzed researcher-supporting GiAs in the life and medical science fields offered by the JSPS, which mainly focuses on basic research. In Japan, there are other public grants to support applied research and/or research for industrialization, such as CREST (Creating REvolutionary technological seeds for Science and Technology innovation) and SAKIGAKE (which promotes individual research to nurture the seeds of future innovation and organize unique, innovative networks), offered by the Japan Science and Technology Agency. The analyses of outcomes supported by such grants may clarify the differential role of public funds to generate ETs in applied research fields.

Geographical differences should also be considered as multiple studies have focused on the situation of grant strategies in developed countries [14]. It is reasonable that domestic culture may make these results specific and, to extrapolate more generalizable results, analyses on the situations in developing countries may be fruitful for discussion/conclusions about the priority of concentrated or dispersed styles of funding.

Finally, funders must be careful about using metrics to make assessments on where to allocate funding. Our index based on EKs and HS-EKs is a metric to evaluate one aspect of scientific productivity and creativity. In general, it is widely accepted to conduct citation analyses of articles and patents to assess the effectiveness of public funds [8, 9, 14]. Thus, combinational analyses with the citation impact from both scientific articles and patents are also valuable in order to reveal the significance of emerging topics toward understanding social innovation and impact from public-grant-supported research. Finally, since this study focuses only on life science and medicine, it would be valuable to investigate other fields to seek common, interdisciplinary mechanisms that generate ETs in science.

## Supporting information

S1 Table Grants-in-Aid investigated in this study. (XLSX)

S2 Table Proportion of new application acceptance. (XLSX)

S3 Table Research fields targeted in this study. (XLSX)
